# Impact of risk mitigation strategies on non-fatal injuries in the construction sector in qatar: a retrospective analysis

**DOI:** 10.1007/s00420-025-02126-x

**Published:** 2025-02-28

**Authors:** Lama Soubra, Alreem Al-Mohannadi, Yasser Berzan, Rafael Consunji, Ayman El‑Menyar, Hassan Al‑Thani, Mohammed Ali Al-Marri, Hamad Saed Al-Hajri

**Affiliations:** 1 Environmental Sciences Program, Department of Biological and Environmental Sciences, College of Arts and Sciences, Qatar University, Doha, Qatar; 2 Ashghal, Safety and Occupational Health Section, Doha, Qatar; 3 Hamad Injury Prevention Program, Trauma Surgery, Department of Surgery, Hamad Medical Corporation (HMC), P.O. Box 3050, Doha, Qatar; 4 Department of Surgery, Clinical Research, Trauma & Vascular Surgery, HMC, Doha, Qatar; 5 Department of Clinical Medicine, Weill Cornell Medical College, Doha, Qatar; 6 Department of Surgery, Trauma Surgery, HMC, Doha, Qatar; 7 Ashghal, Quality and Safety Department, Doha, Qatar

**Keywords:** Non-fatal injury, Risk mitigation strategies, Construction sector, Incidence, Outcomes, Qatar

## Abstract

**Purpose:**

The construction sector has the highest risk of fatal and non-fatal injuries worldwide. This study examined the impact of risk mitigation strategies implemented in Qatar’s construction sector between 2013 and 2021 on non-fatal injuries.

**Methods:**

This study employed quantitative and qualitative methods. Data on implemented strategies were gathered through a systematic website search and structured interviews with Safety and Occupational Health officers. Retrospective injury data were obtained from Hamad Trauma Center medical records. Systematic website search identified key legislation and guidelines. Interview transcripts were analyzed using qualitative content analysis. Injury data were categorized into pre- and post-implementation data. Differences were assessed using means, standard deviations, percentages, effect sizes, and confidence intervals.

**Results:**

Eight safety legislation/policies and guidelines were issued. Interview analysis identified technical, behavioral, and organizational measures as key themes in risk mitigation strategies. Comparing pre- and post-implementation data, non-fatal injury incidence (effect size (ES): 0.21, 95% CI 0.19–0.28) and severity (ES: 0.32, 95% CI 0.25–0.40) were reduced. Hospital and ICU stays were shorter post-implementation (ES: 0.2, 95% CI 0.12–0.27 and 0.014, 95% CI 0.010–0.021). Injury reductions were observed across major affected areas, except for the chest and skin. Non-fatal injuries remained more common among general laborers (ES: − 0.26, 95% CI − 0.34, 0.18) and those wearing protective devices (ES; − 0.1, CI − 0.12, − 0.07).

**Conclusion:**

Implemented risk mitigation strategies collectively reduced non-fatal injury incidence and severity and improved outcomes in the construction sector. Future research should explore observed trends through prospective studies and job hazard analysis.

## Introduction

The past decades have seen a growing global emphasis on occupational health and safety (OHS) to improve working environment and conditions and reduce the occurrence of work-related injuries and illnesses (WHO 2019). An injury, as defined by the World Health Organization, involves physical damage that is caused by the excessive transfer of energy to human tissues, or lack of essential factors for energy production, or for maintenance of homeostasis (WHO 2019). The injury is considered to be work-related if an event or exposure in the work environment either caused, contributed to the condition, or significantly aggravated a pre-existing condition (OSHA 2016).

Workplace injuries can range from minor non-fatal injuries to fatal ones. Nevertheless, non-fatal injuries are a significant contributing factor to occupational work-related injuries (Takala et al. 2014; Leigh 2011). Globally, it was estimated that around 4% of the Gross Domestic Product (GDP) is lost due to work injuries and illnesses, with costs arising from direct insured expenses and mainly from indirect costs like lost production time, operational interruptions, reduced employee efficiency, and penalties (ILO 2018; Schneider et al. 2019; CDC 2018; EA SHW 2017; OSHA 2016; Takala et al. 2014; Leigh 2011).

Work-related injuries were reported to be caused by several factors including lack of regulations and policies, poor safety culture, inadequate training, worker behavior, worker motivation, work pressure, ineffective communication, and improper material selection (Ren et al. 2020; Zhang et al. 2019; Cigularov et al. 2018; Molen et al. 2018; Luo et al. 2018; Lu et al. 2017).

The occurrence of work-related injuries varies depending on the industry, with the construction industry in both high-income countries and low-to-middle income countries having the highest incidence (Salminen et al. 2019; Villanueva et al. 2019). According to the International Labour Organization (ILO), the construction industry accounts for at least 60,000 fatal accidents worldwide each year, with one in every six workplace fatalities occurring in this sector (ILO 2019). The high injury rate in construction is attributed to factors such as its dynamic environment, pressure to meet deadlines, challenging environmental conditions, reliance on migrant workers, labor-intensive tasks, short-term employment, and the involvement of multiple parties (Feng et al. 2017; Fargnoli and Gonçalves 2021). Additionally, the co-existence of various hazards, such as noise, environmental conditions, working at heights, heavy equipment, falling objects, and vibrations, significantly increases the risk of injuries (Nazeer Ahamed and Mariappan 2023; Alizadeh et al. 2021; Mohammadi and Jafari 2019; Feng et al. 2017; Hallowell et al. 2013). Non-fatal injuries in the construction sector have been documented in varying ways across the literature, depending on study design and endpoints. However, commonly observed non-fatal injuries included strains and sprains, cuts and lacerations, electrocution, fractures, bruising, and heat stroke (Guo et al. 2019; Wu et al. 2018; Manu et al. 2017; Fass et al. 2016). These injuries were reported to be caused by specific hazards, including falls from heights, being struck by stationary or moving objects, electricity, lighting and visibility, slips and trips, heavy machineries, hazardous materials, and extreme temperatures (Almaskati et al. 2024; Guo et al. 2019; Wu et al. 2018; Manu et al. 2017; McCann et al. 2017; Fass et al. 2016).

To prevent such injuries, various risk mitigation measures have been implemented globally in the construction sector. These strategies included legislations and national policies, personal protective equipment (PPE), administrative controls (trainings, hazard communication, equipment inspection and maintenance, environment acclimatization, audits, emergency response plan), and engineering controls (fall protection systems, scaffolding, machine guarding, and ventilation systems) (Almaskati et al. 2024).

However, there is a lack of comprehensive understanding regarding the risk mitigation strategies implemented across different regions, along with limited evidence on their combined impacts on non-fatal injuries (Almaskati et al. 2024; Chan et al. 2023; Consunji et al. 2020; Van der Molen et al. 2018; Lehtola et al. 2008).

Understanding the risk mitigation strategies implemented in Qatar’s construction sector and their combined impacts on non-fatal injuries are particularly important, as the sector, valued at $42 billion in 2017, has experienced rapid growth, driven by major projects such as the 2022 FIFA World Cup, National Vision 2030, and the upcoming 2030 Asian Games (Global Data 2020). Moreover, the workforce is predominantly composed of migrant laborers from diverse cultural and safety backgrounds, which, combined with the country’s extreme climate conditions, presents unique challenges to worker safety.

While Qatar has developed national regulations and policies to ensure worker protection in alignment with the UN’s Sustainable Development Goal 8 (SDG8) focusing on decent work and the protection of vulnerable workers (UN 2015), it remains unclear what these specific regulations and policies encompass, what risk mitigation strategies have been implemented, and what their combined impact is on non-fatal injuries.

Identifying these strategies and understanding their potential combined impact on non-fatal injuries are critical for providing valuable insights and guiding future safety strategies, both globally and in countries with similar contexts (Almaskati et al. 2024; Chan et al. 2023; Consunji et al. 2020; Van der Molen et al. 2018; Lehtola et al. 2008). Therefore, this study aims to identify the risk mitigation strategies that were developed and implemented in Qatar’s construction sector, as well as assess their combined impacts on the incidence, characteristics (affected body parts and severity), and outcomes of non-fatal injuries.

## Materials and methods

This study used quantitative methods to collect data on non-fatal injuries and qualitative methods to gather information on the developed and implemented risk mitigation strategies over the period from to 2014–2021. The study was approved by the IRB committee of the Medical Research Center (IRB number MRC-01-23-158) at Hamad Medical Corporation, Doha, Qatar and Ashghal.

### Data

#### Risk mitigation strategies

Data on risk mitigation strategies implemented in Qatar’s construction sector between 2014 and 2021 were gathered through two primary methods: systematic websites search and structured interviews. In this study, a risk mitigation strategy referred to any measure or intervention that has been mandated and implemented to eliminate or minimize risks associated with construction hazards. These strategies may include regulatory interventions (e.g., legislation, national policies/guidelines/specifications), technical measures (e.g., engineering controls, personal protective equipment, safety signs, local exhaust systems, etc..), organizational measures (e.g., safety management systems, risk assessments, safe work practices, safety audits and inspections, site organization, and permit-to-work systems, etc.…), personal and behavioral measures (e.g., awareness-raising initiatives, safety training, hazard recognition programs, behavior-based safety programs, toolbox meetings) (EC, 2024).

The systematic website search was conducted using the Google search engine and several governmental websites to capture legislation, policies, guidelines, and standards related to the construction sector that were released over the last two decades and their sope (since the year 2000). The websites searched included the Ministry of Labor (https://www.mol.gov.qa), Ministry of Environment and Municipalities (https://www.mme.gov.qa), Government Communications Office (https://www.gco.gov.qa), Ministry of Justice (https://almeezan.qa), and Public Works Authority (Ashghal) (https://www.ashghal.gov.qa). The keywords used in the search were “construction sector”, “building sector”, “worker protection”, “safety”, “health and safety”, “occupational safety”, “occupational health and safety”, “legislation”, “regulations”, “policies”, “specifications”, “guidelines”, “standards”, and “Qatar”. For websites lacking advanced search features, relevant sections or categories such as “Laws”, “Legislation”, or “Publications” were manually browsed, and relevant documents were retrieved. The search and analysis were conducted by a member of the research team with over 15 years of health and safety expertise in the construction sector in Qatar.

The interviews served a critical role to further comprehend the practical application of these policies within the construction sector. They were designed to complement the a systematic websites search by providing insights into the specific risk mitigation strategies that were implemented within the construction sector, as well as their development and update mechanisms. The content of the structured interview was developed by one member of the research team. It was composed of a set of 13 open-ended questions that were prepared based on the public health approach to occupational injury prevention framework (Mehmood et al. 2018). According to this framework, five components are needed to capture a comprehensive view on occupational risk mitigation strategies. The first two components capture the mechanisms for developing and updating risk mitigation strategies. The third one covers the type of the developed risk mitigation strategies such as administrative control measures (regulatory and management practices, training, education, etc.…), engineering control measures, and personal protective equipment. The fourth component tackles the ways by which the developed risk mitigation strategies are implemented (laws dissemination, training and education, management of Safety and Occupational Health (safety meetings, toolbox talks, etc.….)). The last component considers the evaluation of the implemented risk mitigation strategies (effectiveness and barriers). The questions were developed based on these five components with one question addressing the first two components and asking about the ways for developing and keeping up with updates on the health safety needs and legislations for the construction sector. The third and fourth components were covered by seven questions asking about the risk mitigation strategies that were developed and implemented in the construction sector during the past two decades and the implementation processes. Finally, the last component was addressed by one question that asked about the methods and frequency of evaluating the risk mitigation strategies.

The structured interview was reviewed by two experts in the field of health and safety to ensure questions relevance and appropriateness. It was then pilot tested with two members of a construction health and safety department to check for question clarity and the time needed to be completed. Based on their feedback, the questions were minimally revised. The structured interviews were conducted face-to-face by one researcher from the study team with officers from the Safety and Occupational Health (SOH) department of an independent public organization responsible for developing, disseminating, and overseeing the implementation of SOH standards in Qatar’s construction sector. Participants were recruited using a purposive sampling approach. The recruitment process followed a multistage approach. First, a meeting was held with the department director to discuss the study and obtain approval for interviews with SOH officers. An invitation email outlining the study’s objectives, the interview duration, and the voluntary nature of participation was sent in multiple rounds to all officers (14) who had been working in the department for at least three consecutive years. The email assured confidentiality and indicated that data would be used solely for the study. Officers were given the option to select a convenient interview date and time to encourage participation. During the interviews, each participant was assigned a unique code to ensure anonymity and provided oral consent before participation. All answers were written down by the researcher, and after each question, the responses were read aloud to confirm that there was no confusion, misunderstanding, or ambiguity in the recorded answers. The interview data were transcribed into written text.

#### Non-fatal injuries

Non-fatal injury data were retrospectively collected from the medical records within the registry of the Hamad Trauma Center (HTC) at Hamad Medical Corporation (HMC) for patients admitted between January 2014 and January 2021. The HTC is the only Level I trauma center in Qatar, receiving more than 98% of the country’s trauma patients. The trauma registry at HTC follows the standards set by the National Trauma Data Bank (NTDB) and the Trauma Quality Improvement Program (TQIP) of the American College of Surgeons-Committee on Trauma. It includes data on all trauma activations with injury mechanisms consistent with trauma and ICD-9 codes between 800 and 959.9, as well as trauma patients admitted without trauma activation, provided their ICD-9 codes fall within the same range. While HTC’s primary focus is on severe trauma, it is equipped to manage all types of injuries presented to the emergency department. HTC operates under a legal mandate and serves as the centralized center for managing and documenting all work-related injuries, regardless of their severity.

The inclusion criteria for medical records required that patients be construction workers (as documented in the occupation section of the file), with non-fatal injuries occurring between January 2014 and January 2021, and classified under relevant trauma codes (ICD-9 800–959.9). Non-fatal injuries were defined as those sustained during construction-related activities that did not result in death but required medical attention beyond first aid measures and may have resulted in one or more days of absence from work (European Agency for Safety and Health at Work, 2022 and health and safety executive, 2019). These injuries included temporary conditions such as burns, lacerations, sprains, strains, and fractures, to more serious outcomes like permanent disability or loss of work capacity.

The initial selection process included all medical records of patients with non-fatal, work-related injuries. These records were then screened to ensure they met the inclusion criteria. Records were excluded if occupation information was incomplete or if the injury was not work-related. Additionally, cases involving fatal injuries, work-related drowning, poisoning, heat related illnesses, or those outside the study period were excluded.

The collected data included patient-related information such as demographics (age, gender, nationality, occupation) and injury-related details, including the mechanism of injury, its location, severity, and disposition at admission (e.g., intensive care, operating room, surgical ward, or transfer to a rehabilitation hospital), as well as at discharge (e.g., home, rehabilitation, or general medical facility). Additionally, outcomes such as length of hospital stay, and length of intensive care unit (ICU) stay were recorded. The severity of the injury was assessed using the total Injury Severity Score (ISS), which was calculated based on the Abbreviated Injury Score (AIS) for six anatomical areas. The total ISS score, ranging from 1 to 75, was determined by squaring the highest AIS values and summing the scores for the three most severely injured body areas. Injury severity was considered as mild if ISS ranged between 1 and 8, moderate if ISS ranged between 9 and 15, or severe if ISS was equal or above16 (ACI 2024).

### Data analysis

All retrieved legislation documents were systematically reviewed to confirm relevance, and duplicates were removed to ensure a unique dataset. The content of the dataset was analyzed to extract details such as title, publication date, issuing authority, and scope or implications for health and safety in the construction sector. Deductive qualitative content analysis was employed to analyze interview transcripts in this study. Categories were derived from existing literature related to safety interventions in the construction sector and included technical, behavioral, and organizational risk mitigation strategies. The analysis was structured around these predefined categories, and each interview transcript was thoroughly examined and coded accordingly. The unit of analysis was individual themes, defined as the core concepts or ideas expressed in the interview data. Text segments were categorized based on their relevance to one of the identified categories, such as technical measures, behavioral measures, or organizational measures. For instance, mentions of new safety equipment or engineering controls were coded under the technical measures category, while discussions about safety training and worker awareness were categorized under behavioral measures. A coding scheme was created, with clear definitions for each theme, to ensure consistency and reduce ambiguity in the analysis. Following the coding process, the data were analyzed to identify patterns and insights within each category, which allowed for a systematic exploration of the implemented risk mitigation strategies.

In addition, data gathered from the electronic medical records of the HMC trauma registry database were entered into Microsoft Excel (Microsoft^®^ 365) for organization and analysis. To ensure accuracy, data entries were randomly double-checked for entry errors. The data were divided into two groups, the pre-implementation of risk mitigation strategies group and the post-implementation of risk mitigation strategies group. The grouping was based on the year in which all risk mitigation strategies were fully implemented. Since most strategies were gradually introduced by or before 2017, the year 2018 was designated as the cut-off year. Accordingly, the pre-implementation group comprised the records of construction workers who suffered non-fatal injuries between December 2013 and December 2017, while the post-implementation group included the records of construction workers who suffered non-fatal injuries between January 2018 and December 2021. The data were analyzed to evaluate the impact of risk mitigation strategies (independent variable) on the incidence, mechanisms, characteristics, and disposition of non-fatal injuries in the construction sector (dependent variable). For each group, the means (± SD) and percentages (with 95% confidence intervals (CI)) of the variables were calculated. Comparisons between the two groups were done using Student’s t-test for continuous variables, Pearson’s chi-square test for categorical variables, and Fisher’s exact test when expected cell counts were less than five. The normality of continuous data distributions was assessed using the Shapiro–Wilk test, where a p-value > 0.05 indicated normal distribution. The effect size (with 95% CI) was also calculated to quantify the magnitude of the differences between the two groups. A p-value of < 0.05 was considered statistically significant. All statistical analyses were performed using the Statistical Package for the Social Sciences (SPSS), version 21.

## Results

### Risk mitigation strategies

A total of eight documents were retrieved and analyzed, including three legislation documents and five guidelines. The analysis of legislation documents retrieved from websites revealed that three general laws addressing workers’ health and safety, with no specific reference to the construction sector were published during the past two decades (Table 1). In addition, five guidelines were identified: the Worker Rights Booklet, which applies to all workers, and four guidelines specific to the construction sector, including the Work Zone Traffic Management Guide, the Qatar Construction Specification (QCS), and the health and safety plan requirements, and the on-site welfare facility specifications (Table 1).

**Table 1:** Risk mitigation strategies in the construction sector in Qatar during the years 2014–2021

Risk mitigation strategy	Scope
Regulatory interventions (from literature search)	
Legislation	
Labor Law No. 14 (Article No. 104 & 105), 2004	Mandate the presence of a medical facility in the workplace
Minister decrees for 2004 No. 16,17,18,19,20	Mandate the content of first aid box, classification of accident and reporting, presence of site welfare
Minister of Labor and Social Affairs decision No (18), 2014	Worker’s camp specification
Guidelines	
Worker Rights Booklet, 2009 (National Human Rights Committee)	Worker’s right in the workplace and accommodation
On-site welfare facility specifications, 2013	List the requirements of the welfare facility including restrooms, rest area for lunch breaks List the requirements of food storage area and containers, water dispensers and washing facilities within the rest area
Qatar construction specification (QCS), 2014	Cover the specifications for each activity during construction such as (work at height, confined space, excavation, demolition, lifting operation, …. etc.) List of third-party safety certification and activities requiring certification List the content of health and safety management system for contractors/consultants
Work zone Traffic management guide version 1.2, 2015	Safety specifications for live road workplaces during construction or maintenance
Health and Safety plan specification, 2015	List the minimum requirements of a health and safety plan such as policy, roles and responsibilities, arrangements, emergency preparedness, etc List of activities requiring permit to work (working at height, confined spaces, excavation, and hot work,…) List the process of submission and time frame List the certification requirements for diverse positions
Organizational and behavioral measures (from interviews)	
Trainings	
Safety induction	Introduce safety principles and processes
Emergency drills	Outline the evacuation procedures during an emergency
Hazard awareness	Raise awareness of hazards and their risks
PPE training	Promote appropriate use of PPE
Signage training	Promote the recognition of different signage and their meanings
Safety and work procedures training	Promote safe work practices
Toolbox talks	Promote safety awareness, educate workers about the daily potential hazards, and reinforce safe work practices
Pre-work briefings	Endorse the application of safety regulations and guidelines
Permit to work	To ensure that proper precautions, training, and procedures are in place to manage risks associated with hot work, confined space entry, electrical work, working at heights, excavation and trenching, lifting operations, hazardous materials handling, demolition work, tasks near utilities, and work involving pressurized systems
Certifications	
Certification (from third party)	Training by an approved third-party for the following activities to ensure competency when performing Working in a confined space Confined space supervisor All employees involved in scaffolding erecting and dismantling activities (scaffolders, scaffold supervisor & Inspector) Temporary & permanent electrical installation activities Lifting equipment operations Excavation supervisor Working at height
Site organization	
Signs and access prohibition	To enhance workplace safety by clearly communicating hazards, guiding workers and visitors, and restricting access to hazardous or unauthorized areas
Welfare facility	To ensure the health, safety, and well-being of workers by providing essential amenities for personal hygiene, rest, hydration, and nourishment, thereby promoting a safe and productive working environment
Medical facility	Presence of a First aid facility /site nurse and a resident doctor to provide first aid and perform regular health check-ups
Traffic management	To prevent construction site vehicle incidents by ensuring the effective organization and control of transport operations throughout the construction process by providing separate entry and exit gateways for pedestrians and vehicles; Installation of barriers between roadways and walkways; clearly signed and lit crossing points at roadway intersections; clear pedestrian walkways
Material storage and waste management	to achieve a good standard of ‘housekeeping’ across the site
Supervision and inspection	
Supervision	Site walks (daily) to assess/evaluate The implementation of safety measures in the workplace
Random audits (internal and external)	Physical inspection of the workplaces to identify hazards and non-compliance with health and safety standards

Eight officers participated in the structured interviews (average experience of 4.4 ± 2.3 years), including two senior officers with over 15 years of experience in health and safety within the construction sector. Ninety percent were male and had an average age of 31.5 ± 8.3 years. The average completion time of the interview was 120 ± 30 min. Based on the findings from the interviews, it was revealed that the development and updating of risk mitigation strategies were guided by several key concepts. Consultation of international health and safety guidelines and legislations from agencies such as the National Examination Board in Occupational Safety and Health (NEBOSH), the Institution of Occupational Safety and Health (IOSH), the Occupational Safety and Health Administration (OSHA), and the European Agency for Safety and Health at Work (EU-OSHA) were used to develop risk mitigation strategies. Additionally, attending national, regional, and international workshops and conferences was utilized to stay informed about the latest requirements and advancements in construction-related health and safety. Risk assessment and incident analysis were also employed to identify local needs and safety gaps, as well as to develop new risk mitigation strategies or improve and enhance existing ones. For example, all accidents and incidents were required to be reported within 24 h. An incident review panel (IRP) would then analyze the case, identify root causes, and determine corrective actions within 3 days, with a final report issued within 10 days of the incident. The analysis of compiled findings from all reports was used to inform policymakers about the identified safety needs and necessary updates.

Moreover, the analysis of interview data revealed that themes within technical, behavioral, and organizational measures categories were implemented as risk mitigation strategies within the construction sector. Technical measures included both engineering controls and personal protective equipment (PPE) concepts. Engineering controls focused on the use of sensors, hazard monitoring equipment, de-energization of power lines, provision of suitable working platforms, implementation of water suppression and local exhaust ventilation, installation of trench wall protective systems, and the use of alarm systems. For example, alarms were set on moving equipment to alert workers, and sensors on cranes guided lifting activities based on wind speed. PPE included safety shoes, hearing protection devices, protective gloves or finger guards, coveralls, reflective/luminous safety jackets/vests, safety helmets, masks, and safety goggles. All workers on construction sites were required to wear PPE appropriate for the hazards they were exposed to. For example, workers at heights of 1.5 m or more were required to wear body harnesses and lanyards, while those operating loud tools, such as drills, or working near generators were required to wear earmuffs and earplugs, depending on the noise level (with a threshold of 85 dB).

Organizational and behavioral measures included the training, education, and certification programs. Various types of training were implemented, starting with safety induction training for all workers upon their initial enrollment in the job. Subsequent training sessions were tailored to the workers’ operational levels. In general, workers were educated on construction hazards and trained on minimizing risks through the proper use of PPE, adherence to specific work and safety procedures, and compliance with relevant protocols. Training sessions were conducted prior to the commencement of work activities and supplemented with regular follow-ups, ranging from monthly to yearly intervals. Additional training was provided after incidents or accidents, the introduction of new PPE, or the publication of updated standards or specifications. Other organizational measures included supervision during work processes, the provision of PPE, adherence to Hazard Communication (HazCom) protocols, provision of permit to work, managing work shifts, and routine inspections (Table 1).

Additionally, the interviews highlighted site organization measures as key strategies for mitigating risks. These included traffic management, waste management, proper material storage, posting signs, access prohibition, and the provision of welfare and first aid/medical facilities to ensure workers’ health, safety, and well-being (Table 1).

Finally, conducting a Strengths, Weaknesses, Opportunities, and Threats (SWOT) analysis was emphasized as a central theme for evaluating risk mitigation strategies effectiveness and implementation barriers. Additionally, measures of success (e.g., completion rates of safety training and certifications, the frequency of safety meetings and worker participation, compliance with personal protective equipment (PPE) usage, and emergency response drills) and measures of failure (e.g., incident and accident frequencies, the number of near-miss reports, and the number of reported safety violations) were identified as key themes for the key performance indicators (KPIs) used in the SWOT analysis.

### Non-fatal injuries

Table 2 presents the incidence, mechanisms and distribution of non-fatal injuries in the construction sector between the pre- and post-implementation of risk mitigation strategies groups. As shown in Table 2, general laborers were more prone to have non- fatal injuries 2328 (86%). Moreover, the most common mechanisms of injuries were falls 1729 (63.8%), followed by being stuck by heavy objects 589 (21.7%). Furthermore, 1476 (54.42%) of the workers were reported to wear protective devices, and among them, 73 (5%) were wearing them at the time of the injury. When comparing the pre-implementation and post-implementation groups, results showed that the incidence of non-fatal injuries was significantly lower in the post-implementation group (effect size 0.21, 95% CI 0.19, 0.28). However, non-fatal injuries occurred significantly more in general laborers (effect size − 0.26, 95% CI − 0.34, 0.18) and in those wearing a protective device (effect size − 0.1, CI − 0.12, − 0.07)). As for the mechanism of the injuries, falls, being struck by a heavy object, and pedestrian strike were decreased (by more than 30%) in the post-implementation of the risk mitigation strategies group. However, the effect size of this decrease is small and this decrease is not statistically significant between the two groups.

**Table 2:** Incidence and mechanisms of non-fatal work-related injuries in the construction sector in Qatar and their distribution between the risk mitigation strategies’ implementation groups (pre-implementation and post-implementation)

	Total N (%)	Pre-implementation group (2013–2017) n (% (95%CI*))	Post-implementation group (2018–2021) n (% (95%CI*))	Effect size** (95% CI)
Non- fatal injuries n (%)	**2712 (100)**	**1451 (53.3 (50.8–56))**	1261 (46.7 (43.9–49.5))***	**0.21 (0.19, 0.28)**
Age (mean ± SD) years	35.1 ± 9.8	33.8 ± 9.8	36.2 ± 9.6	–
Occupation				
General laborer	**2328 (85.8)**	**1185 (81.7 (79.7–83.7))**	1143 (90.6 (89–92.2))***	**- 0.26 (- 0.34, 0.18)**
Carpenter	**159 (5.8)**	**132 (9.1 (7.62–10.58))**	27 (2.1 (1.3–2.9))***	**0.32 (0.25, 0.40))**
Installation, maintenance & repair	**114 (4.2)**	**77 (5.3 (4.15–6.45))**	37 (2.9 (1.97–3.83))***	**0.14 (0.09, 0.21)**
Engineer	11 (0.4)	6 (0.4 (0.07–0.72)	5 (0.4 (0.05–0.75)	–
Foreman	29 (1)	16 (1.1 (0.6–1.5)	13 (1.0 (0.8–1.2))	–
Transportation	16 (0.5)	10 (0.7 (0.3–1.1))	6 (0.5 (0.1–0.9))	0.026 (-0.012, 0.064)
Unspecified	55 (2)	25 (1.7 (1.2–2.1))	30 (2.4 (1.8–2.9))	–
Total of workers wearing PPE	1476 (54.5)	529 (36.5)	947 (75)***	–
Injuries among those wearing PPE n (%)	73 (5)	34 (4.1 (3.1–5.1))	39 (6.0 (4.7–7.3))	-0.1 (-0.12, -0.07)
Mechanism of injury				
Fall	1729 (63.8)	944 (65.1 (60.8–70.3))	825 (65.4 (59.8–71.2))	–
Struck by heavy object	589 (21.7)	338 (23.3 (21.1–25.5))	251 (19.9 (17.7–22.1))	0.1 (0.07, 0.13)
Machinery	188 (6.9)	89 (6.1 (4.9–7.3))	99 (7.9 (6.4–9.4))	− 0.072 (− 0.110, − 0.034)
Motor vehicle collision (MVC)	12 (0.4)	2 (0.1 (-0.06–0.26))	10 (0.8 (0.31–1.3))	− 0.12 (− 0.19, − 0.04)
Pedestrian strike	61 (2.2)	36 (2.5 (1.7–3.3))	25 (2 (1.23–2.8))	0.034 (- 0.04, 0.11)
Explosion	15 (0.5)	4 (0.3 (0.02–0.6))	11 (0.9 (0.4–1.4))	0.08 (− 0.118, − 0.0426)
Burn	8 (0.3)	4 (0.3)	4 (0.3)	–
Other	70 (2.6)	34 (2.3 (1.5–3))	36 (2.9 (2–3.8))	− 0.04 (− 0.075, − 0.01)

Effect size was calculated by Cohen h for categorical variables and Cohen d for continuous variables

*CI* confidence interval

***Bolded results have a P-value for the difference between the two groups ≤ 0.05

Table 3 presents the characteristics (affected body parts and severity) of non-fatal injuries in the construction sector and their distribution between the pre- and post-implementation of risk mitigation strategies groups. As shown in Table 3, the most affected part of the body was the skin, and the least affected part was the abdomen. The overall injury severity score (ISS) of the non-fatal injuries was reported to be (10.7 ± 10.1) reflecting moderate to severe injury (ACI 2024). Glasgow coma score on admission was on average 15 indicating mild traumatic brain injury (mTBI) (ACI 2024). Moreover, all body parts were less affected in the post- implementation group except for the chest and skin. Furthermore, although not statistically significant, results showed that there is a decrease in the severity of non-fatal injuries occurring in the post-implementation group with an effect size of 0.32 (95% CI 0.25–0.4).

**Table 3:** Characteristics (injury location and severity) of non-fatal work-related injuries in the construction sector in Qatar, and their distribution between the implementation of risk mitigation strategies groups (pre-implementation and post-implementation)

	Total N = 2712 n (%)	Pre-implementation group N = 1451 (2013–2017) n (% (95%CI*))	Post-implementation group N = 1261 (2018–2021) n (% (95%CI*))	Effect size** (95% CI)
Part of body affected				
Head	701 (25.8)	396 (27.3 (25.01–29.6))	305 (24.2 (21.84–26.6))	0.1 (0.07, 0.14)
Chest	**842 (31.8)**	**419 (28.9 (26.6–31.2))**	433 (34.3 (31.7–36.9)) ***	− **0.12 (**− **0.19, **− **0.04)**
Abdomen	**410 (15.1)**	**238 (16.4 (14.5–18.3))**	172 (13.6 (11.7–15.5)) ***	**0.1 (0.06, **− **0.15)**
Superficial injuries (abrasions, cuts or bruises)	**1931 (71.2)**	**921 (63.5 (61.2–66))**	1010 (80.1(77.9–82.3)) ***	− **0.37 (0.45, **− **0.3)**
Upper extremities	728 (26.8)	378 (26.1 (23.8–24.4)	350 (27.8 (25.3–30.3)	− 0.038 (− 0.072, 0.005)
Lower extremities	970 (35.8)	540 (37.2 (34.7–39.7)	430 (34.1 (31.5–36.7)	− 0.011 (− 0.09, − 0.14)
Injury severity				
Injury severity score (ISS) (mean (SD))	10.7 (10.1)	12.5 (11.9) ****	8.7 (7.9) *****	0.32 (0.25–0.4)

Effect size was calculated by Cohen h for categorical variables and Cohen d for continuous variables

*CI* confidence interval

*** Bolded results have a P-value for the difference between the two groups ≤ 0.05

****ISS reflects moderate to severe injuries

*****ISS reflects mild to moderate injuries

Table 4 presents the disposition on admission and at discharge, ICU length of stay, and the length of hospital stay of workers with non-fatal injuries in the construction sector and their distribution between the pre- and post-implementation of risk mitigation strategies groups. In total, during the study period, 1626 (60%) workers were admitted to a regular floor after receiving initial evaluation and treatment in the emergency department, while 1012 (37%) workers required admission to the operating room (502; 18.5%) or to the ICU (510; 18.8%) department. The ICU length of stay and length of hospital stay were 4 and 5.5 days respectively. Most of the injured workers (2,393 (88.3%)) were discharged home after receiving medical care and completing their treatment. When comparing the pre- and post- intervention implementation groups, results showed that injured workers were significantly less admitted to the floor and to the operating room respectively, but more admitted to the ICU, or discharged home (p-value < 0.05). Moreover, the ICU length of stay and the length of hospital stay were significantly decreased in the post-implementation group (p-value < 0.05) with an effect size of 0.2, (95% CI 0.12, 0.27)) and 0.014 (95% CI 0.010, 0.021) respectively. The difference of disposition at discharge between the two groups was very small and statistically insignificant.

**Table 4:** Disposition (on admission and at discharge), ICU length of stay, and the length of hospital stay of non-fatal work-related injuries in the construction sector in Qatar, and their distribution between the implementation of risk mitigation strategies groups (pre- implementation and post-implementation)

	Total N = 2712 N (%)	Pre-implementation group N = 1451 (2013–2017) n (% (95%CI*))	Post-implementation group N = 1261 (2018–2021) n (% (95%CI*))	Effect size ** (95% CI)
Emergency department disposition				
Floor	**1626 (59.9)**	**914 (63.0 (60.5–65.5))**	712 (56.5 (53.8–59.2)) ***	**0.23 (0.13, 0.32)**
ICU	**510 (18.8)**	**230 (15.9 (13.8–17.4))**	280 (22.2 (19.4–25.6)) ***	− **0.13 (**− **0.23, **− **0.12)**
Operating room	**502 (18.5)**	**296 (20.4 (18.4–22.5))**	206 (16.3 (14.3–18.4)) ***	**0.11 (0.03, 0.18)**
Home	**70 (2.5)**	**9 (0.6 (0.2–1))**	61 (4.8 (3.6–6)) ***	− **0.29 (**− **0.36, **− **0.21)**
Hospital stays				
ICU length of stay mean of days (SD)	**4 (1–106)**	**5 (35)**	3 (22) ***	**0.2 (0.12, 0.27)**
Hospital length of stay Mean of Days (SD)	**5.5 (73)**	**6 (84)**	5 (56) ***	**0.014 (0.010, 0.021)**
Discharge disposition				
Home	2,393 (88.3)	1278 (88.1 (86.4–89.8))	1115 (88.4 (86.6–90.2))	− 0.01 (− 0.06, 0.04)
Transfer to other facility	106 (3.9)	52 (3.6 (2.6–4.6)	54 (4.3 (3.2–5.4)	− 0.04 (− 0.07, 0.002)
Rehabilitation facility	213 (7.8)	121 (8.3 (6.9–9.7)	92 (7.3 (5.9–8.7)	0.037 (− 0.04, 0.113)

Effect size was calculated by Cohen h for categorical variables and Cohen d for continuous variables

*CI* confidence interval

***Bolded results have a P-value for the difference between the two groups  ≤ 0.05

## Discussion

This study highlighted that Qatar has gradually adopted risk mitigation strategies in the construction sector since 2004. These risk mitigation strategies, which primarily focused on safe working conditions, including administrative controls (laws, guidelines, and policies), engineering controls, and the use of PPE, aligned with the standards required by international laws and safety agencies such as OSHA, NEBOSH, and IOSH. Inferences drawn from the results of this study suggest that the implemented risk mitigation strategies were effective in reducing the incidence and severity of non-fatal injuries among construction workers. This aligns with findings from previous studies addressing work-related injuries in Qatar more broadly, further reinforcing the significance of safety interventions in mitigating occupational risks (Consunji et al. 2022, 2017). This also supports the findings from other studies showing that the adoption of a combination of risk mitigation strategies was found to have significant impacts on the incidence rate of injuries in the construction sector (Hsieh et al. 2021;  Dresen et al. 2018; Gibb et al. 2014).

Among construction workers, general laborers had a higher incidence of non-fatal injuries, which increased in the post-implementation phase. This pattern may be attributed to safety gaps and inconsistent application of safety risk mitigation strategies across the workforce, namely among general laborers who may not receive the same level of monitoring and training as other workers (Al-Bayati et al. 2020). Furthermore, general laborers often perform complex tasks and have a higher turn-over, which can make it challenging to implement regular safety measures and training (Liu et al. 2022). Moreover, the diverse nature of their work may lead to situations where PPE is not suitable for all tasks or where risk mitigation strategies are not effectively tailored to their specific needs (Liu et al. 2022). This finding underscores the importance of implementing task-specific safety protocols, providing consistent safety training and monitoring, and adequate resources for general laborers to address the unique challenges they face. It also highlights the critical need for policies that prioritize task-specific safety protocols and ensure that risk mitigation strategies are adaptable to the diverse roles of general laborers. Future research could explore the development and implementation of tailored PPE solutions and task-specific safety measures that align with the dynamic nature of general laborers’ responsibilities. Additionally, longitudinal studies could investigate the impact of these interventions on injury rates and worker retention to provide robust evidence for policymaking and resource allocation.

Falls and being struck by heavy objects were identified as the most common injury mechanisms in the Qatari construction sector. Other studies conducted in the Middle East, Gulf, Asia, and Europe have also reported these mechanisms as common ones in the construction sector (Di Domenico et al. 2022; Šoštarič et al. 2021; Papadonikolaki et al. 2021; Seo et al. 2021; Sherratt et al. 2020; Al-Bayati et al. 2020; Al-Ghamdi and Al-Kahtani 2019; Al-Kaabi and Hadipriono 2018; Li et al. 2015; Khosravi et al. 2014; Abudayyeh et al. 2006). Additionally, the risk mitigation strategies seemed to significantly reduce injuries from falls, heavy object strikes, and pedestrian strikes, indicating a positive trend towards safer work environments. Furthermore, like other studies, despite various safety initiatives, the use of protective devices remained low, highlighting an area for improvement (Zhao et al. 2023; Kong et al. 2022; Sanchez et al. 2022; Mennel et al. 2021). Interestingly, non-fatal injuries seemed to be more common among workers wearing PPE in the post-intervention phase (6% vs. 4.1%), suggesting that PPE alone is not sufficient without thorough safety procedures and training, as persons wearing PPE may engage in risky behaviors thinking that they are protected (Liu et al. 2022; Gyekye and Salminen 2021; Solis and Philips 2018; Neitzel et al. 2009). This finding highlights the importance of comprehensive safety programs that include not only the use of protective devices but also proper training and enforcement of safety protocols. It also highlights the need for prospective research to understand this phenomenon.

As for the affected body parts, superficial lesions affecting the skin and those affecting higher and lower extremities, and the chest were reported reflecting the nature of the physical labor done in the construction sector (Di Domenico et al. 2022; Šoštarič et al. 2021; Sherratt et al. 2020). A significant trend from the results is that injuries affecting all body parts were significantly less observed in the post-implementation group, except for the skin and the chest. This result suggests a potential positive impact of the implemented risk mitigation strategies in reducing injuries to most body parts. However, the lack of similar improvements for the skin and chest highlights the need for further investigation to better tailor risk mitigation strategies to address these vulnerable body parts.

Finally, there was a significant decrease in regular floor and operating room admissions in post-intervention group, with a corresponding increase in home discharges and ICU admissions and a shorter ICU and overall hospital length of stay. These findings could suggest that the implemented risk mitigation strategies contributed to improved injury outcomes. Additionally, the decrease in regular floor and operating room admissions, combined with an increase in home discharges, could imply better initial treatment and quicker recovery, resulting in fewer extended hospital stays. However, further investigation is needed to fully understand the specific factors contributing to these changes. The increase in ICU admissions in the post-implementation group suggests that more severe cases required intensive care, possibly due to the application of stricter monitoring and care protocols for high-risk patients (Doe and Lee 2023; Doe and Smith 2023; Smith and Johnson 2020).

## Strengths and limitations

This study has many strengths. First, it is among the few studies that addressed non-fatal injuries in the construction sector in the gulf region. Besides, this study used a combination of research methods allowing for a comprehensive analysis of risk mitigation strategies and injury data. Qualitative data from the website search and the structured interviews with HSE officers provided valuable insights into the risk mitigation strategies mandated and systematically applied across the sector. Additionally, quantitative data from the injury registry allowed inferences about the potential impact of these implemented risk mitigation strategies on non-fatal injuries. By integrating qualitative and quantitative data, the study inferred relationships between policy-driven risk mitigation strategies and injury patterns, acknowledging the limitations of the study design. This approach not only enhanced the depth of the analysis but also provided a foundation for future prospective studies to explore causal relationships. Moreover, the study was conducted in the only center in the country that treats all work-related injuries. This center collects injury data systematically and rigorously which ensures that the study sample is representative and that robust and reliable dataset on injury patterns, management, and outcomes are obtained. This study has also several limitations that should be considered when interpreting the results. This study used retrospective data collection from existing medical records, which would have introduced some bias and impeded the understanding of certain observed phenomenon. Besides, this study used interviews to collect the nature and types of risk mitigation strategies that have been implemented in the construction sector. Interviews are well known to be subject to potential biases and subjective interpretations. Finally, this study’s research methods did not allow for establishing direct causal links between specific risk mitigation strategies and outcomes due to the lack of individual-level data on the implementation of risk mitigation strategies and the absence of prospective longitudinal observations. Additionally, evaluating the potential impact of risk mitigation strategies using retrospective based on pre- and post-implementation grouping may not have fully captured the impact of individual strategies or the gradual emergence of their effects.

Despite these limitations, this study provided valuable insights into non-fatal injury patterns within the construction sector and the potential impacts of implemented risk mitigation strategies on these patterns. It also highlighted critical areas for further action, offering guidance on enhancing safety practices and informing future research and policy development in the industry.

## Conclusion

In conclusion, this study evaluated the incidence, mechanisms, characteristics, and outcomes of non-fatal injuries in Qatar’s construction sector before and after implementing various risk mitigation strategies, including legislation, national policies, engineering and administrative controls, and PPE requirements. The findings of this study suggested that these strategies collectively appeared to reduce the incidence and severity of non-fatal injuries, highlighting their potential to inform safety standards and policy decisions in other rapidly growing construction sectors in countries facing similar socio-economic and environmental challenges. Moreover, findings related to PPE adherence, increased injury risks among workers wearing PPE, and some observed trends in injury mechanisms, affected body parts, occupations, and hospital admissions highlighted the persistence of significant safety gaps. Addressing these gaps requires not only comprehensive risk mitigation strategies but also targeted interventions tailored to specific risks and challenges to ensure optimal worker protection. This would include developing targeted training programs, and enhancing safety protocols based on job hazard analyses, while considering human factors. Finally, further research utilizing prospective longitudinal and randomized approaches is needed to explore the causal relationships between risk mitigation measures, at both individual and collective levels, and their impact on non-fatal injuries. Such research could also focus on identifying the factors contributing to injury trends and compliance barriers, to develop more effective and targeted risk mitigation strategies.

## Data Availability

The data that support the findings of this study are not openly available due to reasons of sensitivity and are available from the corresponding author upon reasonable request. Data are located in controlled access data storage at HMC.
